# Immunomodulatory Function of the Tumor Suppressor p53 in Host Immune Response and the Tumor Microenvironment

**DOI:** 10.3390/ijms17111942

**Published:** 2016-11-19

**Authors:** Yan Cui, Gang Guo

**Affiliations:** Department of Biochemistry and Molecular Biology, Cancer Immunology, Inflammation & Tolerance Program, Georgia Cancer Center, Augusta University, Augusta, GA 30912, USA; gguo@augusta.edu

**Keywords:** tumor suppressor p53, *p53* inactivation, tumor microenvironment, immune suppression, inflammation, innate immunity, adaptive antitumor immunity, immunotherapy

## Abstract

The tumor suppressor *p53* is the most frequently mutated gene in human cancers. Most of the mutations are missense leading to loss of p53 function in inducing apoptosis and senescence. In addition to these autonomous effects of *p53* inactivation/dysfunction on tumorigenesis, compelling evidence suggests that *p53* mutation/inactivation also leads to gain-of-function or activation of non-autonomous pathways, which either directly or indirectly promote tumorigenesis. Experimental and clinical results suggest that *p53* dysfunction fuels pro-tumor inflammation and serves as an immunological gain-of-function driver of tumorigenesis via skewing immune landscape of the tumor microenvironment (TME). It is now increasingly appreciated that *p53* dysfunction in various cellular compartments of the TME leads to immunosuppression and immune evasion. Although our understanding of the cellular and molecular processes that link p53 activity to host immune regulation is still incomplete, it is clear that activating/reactivating the p53 pathway in the TME also represents a compelling immunological strategy to reverse immunosuppression and enhance antitumor immunity. Here, we review our current understanding of the potential cellular and molecular mechanisms by which p53 participates in immune regulation and discuss how targeting the p53 pathway can be exploited to alter the immunological landscape of tumors for maximizing therapeutic outcome.

## 1. Introduction

The tumor suppressor p53, as a crucial transcription factor controlling the life and death of cells, has been known to prevent tumorigenesis via inducing apoptosis and senescence [1,2,3,4]. During the past decade, compelling evidence revealed that p53, as a master transcription factor and a crucial sensor of stress, participates in the regulation of a plethora of physiological processes throughout the entire lifespan of the organism, including stem cell state, development, tissue homeostasis, metabolism, and aging [5,6,7,8,9]. Thus, it is not surprising that *p53* dysfunction is linked to dysregulation of stem cell homeostasis, metabolism, autophagy, angiogenesis, migration, and invasion [6,10,11,12], all of which are linked to the hallmarks of cancer [13,14]. Similarly, experimental and clinical observations also suggest that environmentally induced damage and genetic instability are associated with *p53* dysfunction and inflammation [15,16,17]. Because chronic inflammation is a hallmark and a driver of cancer [13,18,19], it is plausible that *p53* dysfunction also contributes immunologically to tumorigenesis and tumor progression by altering host immune responses.

Historically, especially within the first 20 years of p53 research, the connection of p53 with host immune response and regulation had been mostly limited to employing fragments of p53 protein as tumor-associated antigens (TAAs) for tumor vaccines [20,21,22] because many forms of p53 mutation stabilize the p53 protein, resulting in elevated p53 level in tumors [1,3,23,24]. Recently, our studies and those of others have shown that *p53* inactivation/dysfunction alters the immune landscape of the tumor microenvironment (TME) towards pro-tumor inflammation [25,26,27,28], whereas *p53* reactivation or restoration changes the milieu of TME to promote antitumor immunity [29,30,31]. It has been increasingly appreciated that p53 activity potentially regulates host immune function and modulates the immunological landscape of the TME. However, our comprehension of the cellular and molecular mechanisms through which p53 activity regulates host immune response and immune surveillance to tumors is still limited. Here, we review p53-related publications with specific focus on the implication of p53 activity and/or *p53* dysfunction in the host immune response and immunological aspects of tumorigenesis, respectively. We also discuss the potential novel strategies of targeting the p53 pathway for immunotherapy application to improve therapeutic outcome based on existing literature and our unpublished observations.

## 2. *Trp53* Dysfunction and Inflammation—The Non-Canonical and Non-Cell Autonomous Mechanism of Tumorigenesis

It is indisputable that p53 activation-induced apoptosis and senescence is a crucial mechanism of tumor suppression, the so-called autonomous mechanism. Recently, compelling evidence demonstrates that p53 also suppresses tumorigenesis via changing the function and milieu of the transformed cells, which is regarded as the non-cell autonomous mechanism of tumor suppression [32,33]. One such non-cell autonomous mechanism that has been increasingly appreciated is the promotion of chronic inflammation.

It is important to be reminded that, in general, host immune response is an essential defense mechanism against pathogens and/or other environmental stress via an acute surge of innate immune cells, including monocytes, macrophages, dendritic cells (DCs), and natural killer (NK) cells, followed by activation of adaptive immune cells, including T and B cells, and release of effector cytokines and chemokines. The timely termination of activated immune effector cells and cytokines/chemokines when the potential threats are well controlled is as important as the rapid immune activation. Chronic inflammation, on the other hand, differs from the protective acute immune activation in that the unresolved pathogenic stimuli or endogenous stress lead to continued stimulation and recruitment of innate and adaptive immune cells, as well as constant release of pro-inflammatory cytokines/chemokines, all of which skew the milieu towards low-grade persistent inflammation in favor of tissue remodeling/wound healing and suppression of productive immunity. Therefore, chronic inflammation, as one of the hallmarks of cancer, compromises the proper balance of productive immune function and provides a favorable microenvironment for tumor initiation, progression, and metastases [13,16,18,19,34,35,36].

### 2.1. Cellular Components of the Tumor Microenvironment Involved in *p53* Dysfunction-Induced Chronic Inflammation

**Figure 1:**
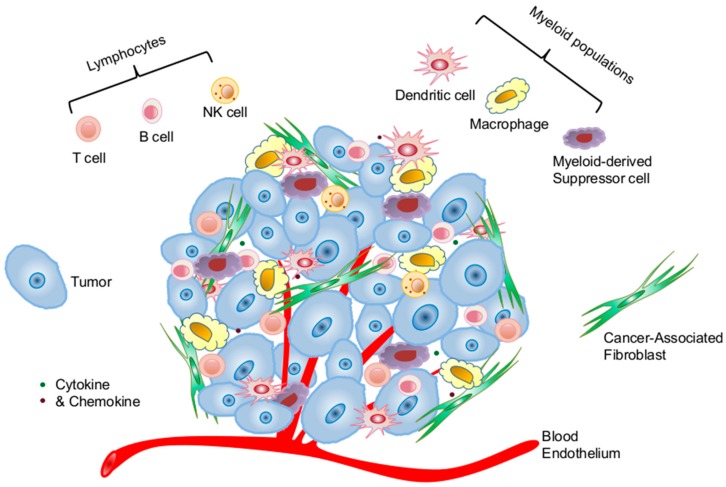
Cellular constituents of the tumor microenvironment that shape tumor immunological landscape. The tumor microenvironment consists of complex cellular and molecular and constituents. The cellular constituents consist of immune cells of hematopoietic origin and stromal cells of non-hematopoietic origin. The immune cell compartment comprises tumor-infiltrating lymphocytes of T, B, and natural killer cells and tumor-associated myeloid populations of dendritic cells, macrophages, and myeloid-derived suppressor cells. The stromal compartment consists of cancer-associated fibroblasts and endothelial cells of the lymphatic and blood vasculature. *Trp53* inactivation has been observed in some of the cellular subpopulations of the tumor microenvironment, which compromises the proper balance of host immunity by promoting chronic inflammation and tumor progression.

Accumulating evidence suggests that tumor progression and metastases are markedly affected by the molecular and cellular constituents surrounding and within the tumor parenchyma, the so-called tumor microenvironment (TME) [37,38,39]. The TME is a highly complex functional ecosystem of tumor and other cellular and molecular components. The cellular constituents of the TME consist of stromal cells (cancer-associated fibroblasts—CAFs, blood, and lymphatic endothelial cells), tumor-infiltrating lymphocytes (T cells, B cells, and NK cells), and myeloid populations (DCs, macrophages, and myeloid-derived suppressor cells) (Figure 1). Many of the tumor-infiltrating immune cells possess immune suppressive function, such as regulatory T cells (Treg), myeloid-derived suppressor cells (MDSC), and type 2 macrophages (M2), which all actively sustain pro-tumor inflammation and immunosuppression [38,39,40,41]. The molecular constituents of the TME consist of extracellular matrix, cytokines and chemokines, and soluble immunosuppressive molecules. All these cellular and molecular components coordinately dictate the immunological landscape of the TME that promotes tumor initiation, progression, and metastasis by creating a chronically inflamed milieu that also protects tumor from immune surveillance and immune destruction [35,42,43].

#### 2.1.1. Cancer Cells of Epithelial Origin

*p53* mutations occur most frequently in solid tumors, i.e., cells of epithelial origin [1,23,24]. Although mutation frequency varies among different tumor types, *p53* mutations occur at a very high frequency in inflammation-associated cancers, such as colon, lung, pancreatic, and ovarian cancers [15,16,44]. This correlation between p53 mutation and inflammation does not explicate the cause-effect relationship. However, early clinical reports of highly elevated p53 activity in pathological tissues of autoimmune diseases, including rheumatoid arthritis (RA) [45,46], ulcerative colitis (UC) [15], and Sjögren’s syndrome (SS) [47], indeed tend to suggest that p53 is a sensor of inflammatory stress [48]. Subsequent clinical studies illustrated that increased p53 expression in the inflamed tissues is associated with somatic dominant-negative *p53* mutations [15,45,47]. Together, these data imply that inflammation-induced p53 upregulation likely serves as a selection pressure for *p53* mutation.

While clinical observations only assist us in establishing potential correlation between *p53* inactivation and chronic inflammation, experimental approaches using *p53^null^* mice allow us to draw a more solid conclusion about the crucial role of *p53* inactivation in amplifying chronic inflammation. Studies by different laboratories reveal that *p53^null^* mice are very susceptible to antigen- and chemical-induced autoimmune diseases [49,50,51], whereas *p53* gene transfer to inflamed tissues alleviates inflammation and autoimmune pathology [52]. Recent results from our laboratory and others further support the notion that *p53* inactivation/dysfunction skews tumor milieu towards pro-tumor inflammation, thereby promoting tumorigenesis and progression [25,26,27]. It is noteworthy that this *p53* inactivation-mediated gain of immunosuppressive function is not restricted to *p53* deletion because the mouse model of *p53* mutation, which mimics human hotspot *p53* mutations, also exemplifies exacerbated tissue inflammation in intestinal epithelial cells, liver, and breast tissues, leading to the development of colon, liver, and breast cancers, respectively [53,54,55]. Thus, these results suggest that while *p53* mutation/inactivation can be induced by environmental stress and tissue inflammation, the resulting inactivation of the p53 pathway in epithelial cells further amplifies chronic inflammation in the TME that likely promotes a vicious cycle of altered immunological milieu that promotes tumor progression and metastasis. Mechanistically, this *p53* inactivation-induced inflammatory capacity of epithelial cells is likely a result of nuclear factor κ-light-chain-enhancer of activated B cells (NF-κB)-mediated production of inflammatory cytokines, which is discussed in the molecular mechanism section.

#### 2.1.2. Cancer-Associated Fibroblasts

Besides the direct pro-inflammatory effects of *p53* mutation in epithelium, early studies indicate that tumor cells also impact the TME via altering the function and properties of adjacent cells, including CAFs. Fibroblasts have long been implicated in chronic inflammation and tumorigenesis by providing the signals for leukocyte recruitment, survival, and retention [56,57,58]. Although our understanding of CAFs on immune regulation is still evolving, recent results suggest that CAFs are somewhat reminiscent of the specialized fibroblastic reticular cells of the secondary lymphoid tissues that are known to be vital in recruiting, organizing leukocytes, and regulating immune responses [59,60,61,62,63,64].

Interestingly, *p53* mutations are detected in the CAFs of highly inflamed cancers that maintain an intact p53 pathway [65,66,67]. Moreover, these *p53* mutations in CAFs are associated with an increased rate of tumor metastasis and poor prognosis [65,66,68]. Mechanistically, it is demonstrated that the pro-tumor and pro-inflammatory effects of *p53*-inactivated CAFs is mediated through enhanced production of cytokines and chemokines, including C-X-C motif chemokine ligand 12 (CXCL12)/stromal cell-derived factor 1 (SDF-1) and interleukin (IL)-6, which markedly affect both immune cell composition and function in the TME [25,32,57,58,69,70,71]. Furthermore, recent studies in our laboratory illustrate that fibroblastic stromal network in the TME of *p53^null^* mice enhances MDSC proliferation and accumulation, thereby exacerbating immunosuppression and tumor progression [25]. It is also reported that *p53* dysfunction in CAFs serves as a selective pressure for the transformation of adjacent epithelial cells [33], which underscores the importance of functional p53 in other components of the TME in suppressing tumor initiation, progression, and metastases. More comprehensive studies are needed to further delineate the multiple pathways that CAFs are involved in for regulating the immune landscape of the TME.

#### 2.1.3. Immune Cells

Immune cells constitute an important cellular compartment of the TME (Figure 1), although their abundancy varies greatly among different tumor types and from individual to individual. It is now increasingly appreciated that their abundancy, especially subset composition, is associated with disease progression and clinical prognosis [72,73,74,75]. Productive antitumor immunity heavily relies on the number and quality of activated effector T cells, whereas the existence and high frequency of immunosuppressive Treg and MDSCs are detrimental for immune-mediated tumor control.

Clinically, *p53* mutations are less frequently detected in immune cells. Even in hematopoietic malignancies that are associated with *p53* mutations, the potential effects of *p53* dysfunction/inactivation on host immune regulation have been largely overlooked. Thus, our understanding and assumption of immune cell *p53* inactivation in altering host immune response and function is derived from experimental observations using *p53^null^* mice. For instance, Zheng et al. showed that *p53* inactivation enhanced the production of inflammatory cytokines IL-1, -6, and -12 by macrophages [50]. Furthermore, experimental results from our laboratory and others demonstrated that in addition to overall enhanced IL-6 production, *p53* inactivation in T cells also enhanced their differentiation to T helper Th17 cells [26,50,51]. Recent studies also demonstrated that *p53* inactivation compromises Treg differentiation, which shifts the balance between tolerance and inflammation towards inflammation [76,77]. Because Th17/IL-17 activity has been linked to inflammation, autoimmunity, and tumorigenesis [78,79,80], these results verify that *p53* inactivation in immune cells augments inflammation-induced tumorigenesis via multiple pathways, such as enhancing production of inflammatory cytokines and chemokines, promoting the differentiation and function of Th17 cells, and suppressing the differentiation of Treg. This crucial role of p53 in suppressing chronic inflammation is also confirmed by gene delivery experiments showing that *p53* gene transfer suppresses inflammation and autoimmunity [17,50].

It is noteworthy that, clinically, functional inactivation of the p53 pathway in immune cells or other cellular subsets may occur in the absence of *p53* mutation. It has been shown that overexpression of the natural p53 inhibitory protein mouse double minute 2 homolog (MDM2) or expression of certain viral proteins upon viral infection, such as human papillomavirus (HPV) E6 and human T-lymphotropic virus 1 (HLTV-1), also lead to inhibition of p53 activity, which has been linked to tumorigenesis [81,82,83,84]. Interestingly, HTLV-1 infection appears to be associated with increased development of autoimmune diseases, including RA, SS, and systemic lupus erythematosus (SLE) [85], although it is yet to be determined whether this HTLV-1-induced chronic inflammation is at least partially resulted by *p53* dysfunction in immune cells.

### 2.2. Molecular Pathways that Link *p53* Dysfunction and Inflammation

#### 2.2.1. NF-κB Hyperactivity

NF-κB, as a crucial transcription factor, is implicated in inflammation and tumorigenesis. Interestingly, it has been frequently reported in various experimental systems that NF-κB activity often shows a negative correlation with that of p53 [17,86,87]. Molecular studies defining the underlying mechanism of the reciprocal activities between p53 and NF-κB suggested that it is likely caused by their competition for the limited transcription coactivator p300 and the cAMP response element binding protein (CREB)-binding protein [17,86,87]. Moreover, recent studies demonstrate that p53 can also suppress the NF-κB pathway via directly inhibiting the promoter activity of NF-κB subunit p65 or indirectly repressing the activity of an IκBα kinase, IKKα [87,88,89]. In agreement with this notion, our study and those of others also demonstrated that *p53* inactivation causes hyperactivity of NF-κB pathway in *p53^null^* T cells, macrophages, and intestinal epithelium [26,50,55,89]. Therefore, highly activated NF-κB pathway in cancer cells, stroma, and immune cells all lead to elevated production of inflammatory cytokines and chemokines, thereby promoting chronic inflammation and tumorigenesis [26,50,55,89].

Nevertheless, the reciprocal activation of p53 and NF-κB pathways is likely context dependent because under certain circumstances co-activation of p53 and NF-κB was also observed [17,90]. In a recent study involving human macrophages, it was demonstrated that p53 and NF-κB co-regulate IL-6 production [90]. Additionally, senescent cells with highly activated p53 pathway secrete numerous inflammatory cytokines, chemokines, growth factors, and other soluble proteins via activating the NF-κB pathway [91,92].

#### 2.2.2. Signal Transducer and Activators of Transcription Pathways

Signal transducer and activators of transcription (STAT) is a group of crucial transcription factors that instigate signals of cytokines, chemokines, and growth factors to biological function. Various STAT molecules respond to different environmental cues during various biological events. Among them, STAT3 hyperactivity has been shown to be crucial in the development of myeloid-suppressor cells and inflammation-induced tumorigenesis [36,93,94,95,96]. Interestingly, early studies suggested that p53 activity suppresses that of STAT3 so that *p53* inactivation leads to STAT3 activation [97]. Our results of *p53* inactivation-associated autoimmune mouse model revealed that hyperactivated STAT3 and NF-κB pathways in mouse T cells are drivers of Th17 differentiation and autoimmune pathology [26]. Furthermore, the study by Zheng et al. using *p53^null^* in an autoimmune disease model revealed that *p53* inactivation in macrophages resulted in a hyper-responsive STAT1 pathway, leading to enhanced production of inflammatory cytokines [50]. Besides the supportive roles of STAT3 in Th17 differentiation in *p53^null^* mice, it is also reported that p53 activation-induced suppression of Th17 differentiation is mediated through activation of STAT5 [76]. Therefore, p53 activity regulates host immune response partially through modulating the balanced activity of various STAT pathways.

#### 2.2.3. Other Immune Regulatory Molecules and Pathways

Similar to previous reports of *p53* inactivation-promoted production of inflammatory molecules, such as IL-6, cyclooxygenase 2 (Cox-2), and inducible nitric oxide synthase (iNOS) via NF-κB and STAT pathways [16,17,86], *p53* inactivation also enhances the production of macrophage migration inhibitory factor (MIF), a pro-inflammatory cytokine that promotes inflammation via the NF-κB pathway [98,99,100,101]. Recently, the effects of *p53* dysfunction/mutation-induced inflammatory and/or immunosuppressive function have been broadened to the upregulation of programmed death-ligand 1 (PD-L1) via microRNA miR34 [102]. Furthermore, p53 missense mutant binds and inhibits another tumor suppressor disabled homolog 2-interacting protein (DAB2IP) to promote inflammation through activation of NF-κB [44,52].

Collectively, these experimental and clinical data exemplify that *p53* dysfunction in tumors or other cellular populations of the TME also promotes tumor progression and metastasis via enhancing chronic inflammation. This process involves considerable modulations of molecular and cellular composition, as well as tissue structure, reminiscent of tissue remolding and wound healing, which controls the immunosuppressive nature of the TME [13,35,38,39,40,41,103].

## 3. Targeting the p53 Pathway in the Tumor Microenvironment for Improving Systemic Antitumor Immunity

As discussed earlier, acute burst of immune activation, different from chronic inflammation, supports productive and protective immunity. Effective immune-mediated tumor eradication requires tumor antigen-specific, systemic, and durable T cell-mediated adaptive immunity [104,105,106,107]. Because *p53* inactivation leads to pro-tumor chronic inflammation and immunosuppression in the TME, we hypothesize that p53 activation or restoration in the TME reverses immunosuppression and reshapes the immunological landscape to support productive antitumor immunity for better tumor control. In fact, genetic manipulations using mouse models demonstrated that p53 reactivation in p53-deficient tumors induced tumor apoptosis and/or senescence, which resulted in recruitment and activation of innate immune cells, including NK cells and macrophages, for the clearance of the apoptotic or senescent tumor cells [30,31]. However, it is largely unknown whether p53 activation in the TME also promotes tumor-specific T cell activation. Based on the existing reports of potential cellular and molecular mechanisms of p53 activation in enhancing innate immunity, our knowledge of the cooperativity between innate and adaptive immunity, and some of our unpublished observations, we discuss here the contribution of various cellular and molecular pathways to p53 activation-mediated immune activation and potential development of targeting the p53 pathway as a novel immunotherapy strategy for cancer control and clearance.

### 3.1. Effects of *p53* Activation on Innate Immunity

As the first line of host defense to pathogenic and environmental insults, innate immune cells are activated almost instantaneously upon encountering pathogenic threats to kill and engulf the invaders in a non-antigen-specific manner. In general, the activation is initiated by the stimulation of Toll-like receptors (TLRs) on the innate immune cell surface through pathogen-associated molecular pattern (PAMP) or internal stress-related damage-associated molecular pattern (DAMP) signals, such as single-stranded DNA, double-stranded RNA, and bacterial-like DNA fragments containing CpG motifs. This PAMP or DAMP pathway-mediated activation of innate cells results in their enhanced type I interferon (IFN) production, which further activates the myeloid cells for their activation of adaptive immunity. Recent studies also showed that radiotherapy-induced DNA damage resulted in elevated production of type I IFN, which stimulated tumor antigen process and presentation by DCs and subsequent activation of adaptive antitumor immunity [108].

Interestingly, recent compelling evidence suggests that therapeutic efficacy of radio- and chemotherapy, both involving DNA damage and activation of the p53 pathway, is largely dependent on their capacity of eliciting antitumor immunity [109,110,111,112]. Mechanistically, this process is first initiated by therapy-induced tumor immunogenic cell death (ICD), usually characterized by three hallmark markers: exposure of an endoplasmic reticulum (ER) protein, calreticulin (CALR), at the cell surface, release of the chromatin protein high-mobility group box 1 (HMGB1), and ATP to the extracellular space [109,111]. Extracellular exposure of these molecules leads to activation of innate immune cells via various pathways, such as TLR-4 and purinergic receptors [92,113]. Moreover, it has also been reported that p53 activation directly enhances the expression and function of TLR-3 and/or -8 in human cancers, epithelial cell lines, lymphocytes, and type I alveolar cells [27,114,115]. Furthermore, a recent study revealed that p53 by direct binding to the promoter region of IL-12 enhances DC function and capacity of promoting adaptive antitumor immunity [116].

Besides myeloid cells, NK cells potentially can be activated by DNA damage-induced upregulation of UL16 binding protein 2 (ULBP2), a natural killer group 2D (NKG2D) ligand, which greatly enhance NK-mediated tumor elimination [117,118,119,120,121].

Together, these results suggest that p53 activation likely promotes the activation of innate immunity via multiple molecular pathways. Based on existing literature and our unpublished observations, we propose that some of the immunostimulatory effects of conventional radio- and chemotherapy are mediated through p53-associated TLR and IFN pathways. It is yet to be verified clinically whether therapy-induced p53 activation contributes to the induction of antitumor immunity.

### 3.2. Effects of *p53* Activation on Adaptive Immunity

Recent advances in tumor immunology and immunological assessment of the therapeutic effects of conventional radio- and chemotherapy suggest that the activation of the adaptive antitumor immunity is indispensable for their therapeutic benefits [109,110,111]. Because innate immunity serves as a crucial activator of the adaptive immune responses, p53 activation-mediated stimulation of innate immunity, especially DC activation, will also promote adaptive immunity.

On the other hand, our understanding of the direct effects of p53 activation on T and B activation is still very limited. Early studies showed that p53 activation is readily detected in mitogen-stimulated or T cell receptor (TCR) ligation-activated murine and human T cells [122,123]. However, for total T cell population, this elevated p53 activity does not appear to impose detrimental effects on their survival or function [45,46,47,124]. Only until recently, it was believed that p53 activation during T cell activation serves as a selection factor for eliminating non-productive T cells that are stimulated by IL-2, but not fully activated by antigen-specific TCR ligation [125]. It is clear that more in-depth studies are required to advance our comprehension and appreciation of the regulatory role of p53 in adaptive immunity.

Altogether, p53 activation can directly enhance T cell antitumor immunity by selecting effectively activated effector T cells and indirectly by stimulating innate immune responses that enhance the tumor antigen update, presentation, and activation of myeloid cells for promoting activation of tumor antigen-specific adaptive immunity.

### 3.3. Potential Application of Targeting the p53 Pathway for Harnessing Host Antitumor Immunity

Because *p53* mutation/inactivation is one of major causes of cancer, targeting the p53 pathway for direct tumoricidal effects has been an important strategy for cancer treatment [126,127,128]. Immunologically, activation of the p53 pathway in the TME elicits both innate and adaptive immunity and, thus, can be exploited to harness antitumor immunity for additional therapeutic gains. Here, we will focus our discussion on approaches that directly activate the p53 pathway via pharmacological activators.

**Figure 2:**
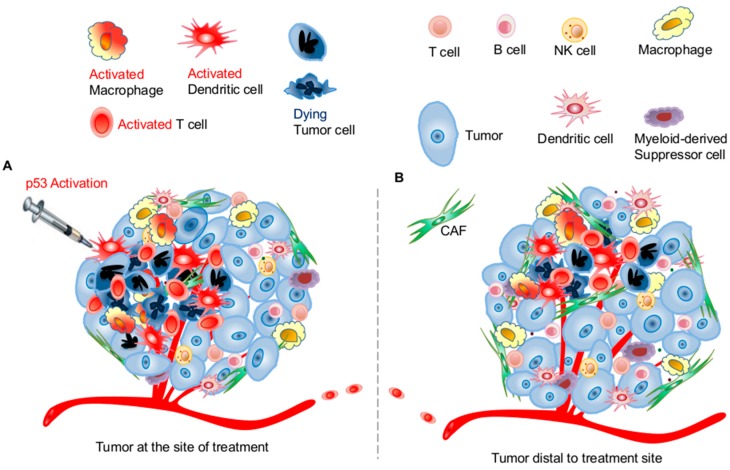
Targeting the p53 pathway in the tumor microenvironment (TME) to activate antitumor immunity for systemic tumor control. Local activation of the p53 pathway in the TME can be achieved via intratumoral injection of specific p53 activators (**A**). This induces tumor immunogenic cell death and subsequent activation of macrophages and dendritic cells at confined areas around the site of injection. Activated macrophages and DCs update and present tumor antigens to activate T cells that kill more tumor cells (**A**). This T cell-mediated tumor killing further amplifies the activation events of macrophages, DCs, and T cells that execute and expand tumor killing around the injection site (B). Importantly, activated T cells travel in circulation through blood and lymphatic vasculatures to find and kill additional tumors distal to the site of injection (**B**). Activated systemic adaptive immunity via local p53 activation in the TME supports systemic tumor control and regression. CAF: cancer-associated fibroblasts.

It is noteworthy that current approaches of targeting the p53 pathway mostly focus on activating or restoring p53 function in tumor cells. As discussed above, p53 activation also greatly impacts the function and survival of immune cells within the TME. Because most immune cells are more sensitive to p53 activation-induced cell death than tumor cells as shown in a previous report [129] and our unpublished observation, targeted p53 activation as an immunotherapy approach will require different dosing and delivery strategies. Specifically, p53 activation-elicited antitumor immunity does not rely on a global or uniform activation of the p53 pathway throughout the entire TME. In fact, local p53 activation that sufficiently induces tumor death and activation of dendritic cells and macrophages in the confined area of the TME can be effective in amplifying T cell-mediated tumor antigen-specific antitumor immunity (Figure 2). These in situ activated tumor antigen-specific T cells will circulate systemically to provide protection and to reach and eliminate distal metastatic tumors (Figure 2). Overall, *p53* reactivation or restoration reverses the immunosuppressive landscape of the TME and promotes antitumor immunity for better tumor control.

#### 3.3.1. Small Molecule-Based Therapy Targeting the p53–MDM2 Axis

The E3 ubiquitin ligase MDM2 is a natural, crucial p53 inhibitor. Thus, a pharmacological inhibitor of MDM2, such as nutlin-3a, activates wild-type p53 [130,131]. Similarly, reactivation of p53 and induction of tumor cell apoptosis (RITA) also induces p53 activation by binding HDM2 human double minus-2, the human analog of MDM2 (HDM2), [132,133]. Thus far, most of the published studies focus on MDM2-mediated tumor cell apoptosis. Interestingly, recent reports demonstrate that nutlin-3 also regulates host immune response by activating antigen-presenting cells, including dendritic cells [134,135], thus suggesting that activating the p53 pathway can also be explored for its immunomodulatory effects to improve therapeutic outcome. In fact, recent unpublished results from our laboratory demonstrated that intratumoral local delivery of nutlin-3a induced ICD of tumors that activate dendritic cells and macrophages, which subsequently activate tumor-specific cluster of differenctiation (CD) 8 T cells. These activated tumor-specific T cells not only eliminated tumor cells at the site of nutlin delivery, but also resulted in regression of tumors distal to the nutlin-injection site (Figure 2).

#### 3.3.2. Restoration of Mutant p53 to Wild-Type Configuration and Function

Because many p53 mutants are associated with conformational changes that hinder their DNA binding and transactivation capacity, small molecules other than MDM2 inhibitors are needed to restore the function of *p53* mutants. p53 reactivation and induction of massive apoptosis-1 (PRIMA) and mutant p53 reactivation and induction of rapid apoptosis (MIRA-3) are representative molecules that convert mutant p53 to restore wild-type function [136,137]. Even though the immunoregulatory property of these pharmacological activators has yet to be explored, given their well understood mechanism of action, it is believed they will broaden the application of targeting the p53 pathway for immunological gains in tumors incurring *p53* mutations. Considering the observed non-overlapping *p53* mutations in either tumors or stromal compartment of the TME, such as tumor or CAFs, we propose that combination of p53 reactivators with MDM2 inhibitors will broaden the spectrum of p53 activation in the TME regardless of the *p53* status, thereby likely representing a more effective approach for promoting antitumor immunity and overcoming tumor-induced immune tolerance.

#### 3.3.3. Combining p53-Activation Therapy with Other Active Immunotherapy to Improve Therapeutic Efficacy

Clinically and experimentally, compelling evidence demonstrates that effective cancer therapy requires multipronged combinational approaches that not only debunk tumors, but also elicit strong antitumor immunity, which is otherwise unachievable by monotherapy of surgery, radiation, chemotherapy, or even single-pronged immunotherapy [109,138,139,140]. One of the crucial factors that dictates immunotherapy outcome is efficient recruitment of immune effectors, especially activated tumor-specific CD8 T cells, to the TME. Given the diversity of immune cell infiltration in different tumors and individuals, it is important to employ other potent immunotherapy approaches for enhancing recruitment of immune cells and for maintaining CD8 effector T cell function at the time of p53 activation-based immunotherapy.

Pharmacological activation of the p53 pathway in the TME represents a new and exciting immunological strategy to reverse immunosuppression of the TME and to promote systemic, lasting antitumor immunity for improved tumor control. Certainly, more in-depth mechanistic and preclinical studies are warranted before their broader clinical applications can be fully appreciated and developed.

## 4. Conclusions

During the past 30 years, our understanding and appreciation of the complexity of the p53 pathway and its regulation in myriad biological processes have been greatly enriched. Nevertheless, our comprehension of p53 in all aspects of physiological processes is far from complete. From an immunological aspect, our knowledge of its participation in host immune surveillance and tumor immune evasion is limited. Compelling evidence supports a crucial immunological consequence of *p53* dysfunction in tumorigenesis and tumor progression. It is, therefore, imperative that targeting the p53 pathway is incorporated into an active immunotherapy approach for maximal therapeutic benefits, which certainly relies on our better comprehension of the specific mechanisms/pathways through which p53 actively regulates host immune responses and the immunological landscape of the tumor microenvironment.
